# Monitoring Online Discussions About Suicide Among Twitter Users With Schizophrenia: Exploratory Study

**DOI:** 10.2196/11483

**Published:** 2018-12-13

**Authors:** Yulin Hswen, John A Naslund, John S Brownstein, Jared B Hawkins

**Affiliations:** 1 Department of Social and Behavioral Sciences Harvard TH Chan School of Public Health Harvard University Boston, MA United States; 2 Informatics Program Boston Children's Hospital Boston, MA United States; 3 Department of Global Health and Social Medicine Harvard Medical School Boston, MA United States; 4 Department of Pediatrics Harvard Medical School Boston, MA United States; 5 Department of Biomedical Informatics Harvard Medical School Boston, MA United States

**Keywords:** schizophrenia, social media, suicide, Twitter, digital technology, mental health

## Abstract

**Background:**

People with schizophrenia experience elevated risk of suicide. Mental health symptoms, including depression and anxiety, contribute to increased risk of suicide. Digital technology could support efforts to detect suicide risk and inform suicide prevention efforts.

**Objective:**

This exploratory study examined the feasibility of monitoring online discussions about suicide among Twitter users who self-identify as having schizophrenia.

**Methods:**

Posts containing the terms suicide or suicidal were collected from a sample of Twitter users who self-identify as having schizophrenia (N=203) and a random sample of control users (N=173) over a 200-day period. Frequency and timing of posts about suicide were compared between groups. The associations between posting about suicide and common mental health symptoms were examined.

**Results:**

Twitter users who self-identify as having schizophrenia posted more tweets about suicide (mean 7.10, SD 15.98) compared to control users (mean 1.89, SD 4.79; t_374_=-4.13, *P*<.001). Twitter users who self-identify as having schizophrenia showed greater odds of tweeting about suicide compared to control users (odds ratio 2.15, 95% CI 1.42-3.28). Among all users, tweets about suicide were associated with tweets about depression (*r*=0.62, *P*<.001) and anxiety (*r*=0.45, *P*<.001).

**Conclusions:**

Twitter users who self-identify as having schizophrenia appear to commonly discuss suicide on social media, which is associated with greater discussion about other mental health symptoms. These findings should be interpreted cautiously, as it is not possible to determine whether online discussions about suicide correlate with suicide risk. However, these patterns of online discussion may be indicative of elevated risk of suicide observed in this patient group. There may be opportunities to leverage social media for supporting suicide prevention among individuals with schizophrenia.

## Introduction

Individuals living with schizophrenia experience elevated risk of suicide compared to the general population [1]. Adults with schizophrenia are nearly four times as likely to die from suicide when compared to adults in the general population (all-causes standardized mortality ratio 3.9, 95% CI 3.8-4.1) [2]. Lifetime risk of suicide among individuals with schizophrenia ranges from 5% to 13%, representing a leading cause of mortality in this group [1,3]. There is urgent need for novel approaches to detect suicide risk among individuals with schizophrenia.

Social media platforms have emerged as important digital monitoring tools capable of facilitating the detection and tracking of numerous diseases and public health concerns [4]. A growing number of studies have highlighted the feasibility and promise of using popular social media for monitoring online conversations about suicide and for potentially detecting those at risk of suicide [5]. Several studies have used Twitter data to characterize suicide-related conversations [6] and to monitor suicide risk [7,8]. An exploration of Tumblr posts found that conversations about suicide were frequently shared together with content about self-harm and depression [9]. Another study investigated the psychological characteristics of social media users in China who posted conversations about suicide on the Weibo microblogging platform [10].

Research has shown that studying social media activity can yield valuable public health insights about serious mental disorders such as schizophrenia. For example, data captured from Facebook was used to characterize awareness of schizophrenia across the United States [11], while another study found that conversations about schizophrenia on Twitter were often negative, suggesting the presence of social stigma [12]. Numerous studies have also demonstrated that individuals living with schizophrenia use popular social media at comparable rates as the general population [13,14]. Further, individuals with mental illness appear to use social media to share their illness experiences or seek advice from others with similar conditions [15,16]. A series of recent studies have identified unique patterns of communication on Twitter among users who self-identify as having schizophrenia, as reflected by linguistic differences, compared to control users [17,18]; changes toward more positive sentiment following self-disclosure of mental illness on social media [19]; and greater use of conversation terms about mental health symptoms compared to control users [20].

In addition, social media such as Twitter may be especially valuable for monitoring suicide risk among individuals with schizophrenia as these platforms offer unique opportunities to engage young adults. For instance, on average, Twitter users tend to be younger compared to the overall population [21]. This is highly relevant because suicide mortality among people with schizophrenia is greatest among younger adults, where individuals 20-34 years of age are over five times as likely compared to young adults from the same age group from the general population to die due to suicide (all-causes standardized mortality ratio 5.3, 95% CI 4.9-5.7) [2]. A literature review found that risk of suicide and self-harm is also highly prominent among young individuals considered at ultra-high risk of developing psychosis [22]. As well, there is increasing recognition that individuals with schizophrenia are heavy users of social media and show unique communication patterns on these digital platforms. This combination highlights the potential to leverage social media for detecting suicide risk and informing suicide prevention efforts in this at-risk patient group. However, less is known about whether people with schizophrenia talk about suicide on social media.

An important first step toward developing strategies to use social media for supporting the detection of suicide risk among individuals with schizophrenia is to better understand how this target population talks about suicide on popular social media. Therefore, in this exploratory study, our aims were to (1) investigate the frequency of online communications about suicide among Twitter users who self-identify as having schizophrenia compared with a control group of typical Twitter users; (2) characterize the timing of tweets about suicide among Twitter users who self-identify as having schizophrenia compared with a control group of typical Twitter users; and (3) determine whether discussion about other common mental health symptoms, including depression or anxiety, is predictive of online discussions about suicide. We hypothesized that Twitter users who self-identify as having schizophrenia would be significantly more likely to post tweets containing suicide terms when compared to Twitter users from the general population, thereby reflecting the elevated risk of suicide observed among individuals with schizophrenia in real-world settings.

## Methods

### Data Collection

All data analyzed in this study was publicly available and was collected from the Twitter social media platform. Twitter is a popular microblogging platform where users post short statuses called “tweets” that contain a maximum of 140 characters—since 2018, this has increased to a maximum of 280 characters for each tweet. It is estimated that the more than 330 million active Twitter users post over 500 million tweets per day [23,24]. This highlights an immense source of unsolicited data with exciting potential to study various aspects of human behavior and monitor health conditions including mental illness [25]. Specifically, we selected this social media platform for this study because it has previously been used for conducting research on several different mental health conditions, including depression, bipolar disorder, and posttraumatic stress disorder [26]. Importantly, data captured from Twitter has been used in research characterizing online discussions and attitudes about schizophrenia [12,27], exploring linguistic markers of schizophrenia [17,18], and supporting efforts to detect individuals with schizophrenia [18,19]. Lastly, Twitter users tend to be younger compared to the overall population [21], which is especially important given the elevated suicide risk among young persons with schizophrenia [2]. Therefore, given that Twitter can achieve widespread reach, and that we can expand on existing related work, we determined that Twitter would be an ideal platform to potentially serve as an effective digital tool for monitoring risk of suicide among people with schizophrenia.

As popular social media platforms have emerged as an important source of user-generated content that can yield valuable insights for public health research, the ethical considerations with analyzing and disseminating this data have received greater attention [28,29]. While there remains a lack of consensus over best practices for using Twitter data in academic research [30], there is ongoing discussion surrounding concerns related to privacy, confidentiality, and informed consent [31]. To minimize potential risks, we ensured that all data collected in our study was available in the public domain. However, additional ethical considerations are warranted, especially in the context of socially stigmatizing health conditions such as mental illness [32]. For example, disseminating user-generated content collected on Twitter could potentially place an individual at risk of harm [30] because sensitive health information, such as mental illness diagnosis or symptoms, could be made identifiable in ways that were not intended by the original user who posted the content online [33]. Therefore, to further protect the identity of the Twitter users whose data we examined in this study, we removed all usernames and identifiable details from the content that they posted online. Lastly, we do not report any specific tweets that could be used to identify the original Twitter user who posted the content online, as this is an important concern that has been discussed extensively in recent literature on the ethics of using Twitter data for research [30]. In this study, we retrieved data from Twitter’s public application programming interface over a 200-day period from January 5, 2016, to July 23, 2016. Given that we only used publicly available online data in this study, ethical review was not required.

### Twitter Users and Characteristics

We identified a convenience sample of 250 Twitter users who explicitly self-identified as having a schizophrenia spectrum disorder in their profile or in a tweet. For example, the users’ profiles could indicate “person living with schizophrenia” or “I have schizophrenia diagnosis,” while a tweet could mention “this is how I manage my schizophrenia” or “I was just diagnosed with schizophrenia.” We modeled our data collection methods on prior studies that have used the Twitter platform for generating a convenience sample of users with publicly available accounts who self-identify as having a schizophrenia spectrum disorder in their profile or in a post or tweet [18,34]. To identify the sample of Twitter users with schizophrenia, we searched Twitter using the following terms: *schizophrenia*, *schizoaffective*, *schizotypal*, and *psychosis*. We then confirmed the self-reported schizophrenia diagnosis by having one researcher generate this initial list of Twitter users and then a second researcher check the details for each Twitter user on the list to ensure correct identification of users with a self-reported schizophrenia spectrum disorder.

To create a general population comparison group, we used the *GET statuses/sample* feature from the Twitter Developer Platform to collect a random sample of all publicly available tweets [35]. Then, two research assistants manually screened these tweets to confirm that the tweet belonged to a real person (ie, not a bot or spam account), was from a normal user (ie, not a company or corporation), and was in English. This process was intended to ensure that Twitter users included in the control group were real Twitter users. To minimize the risk of selecting any bot or spam users, both research assistants had to be in agreement of a Twitter user on each of these three criteria. We excluded any Twitter users where there was disagreement. Our goal was to create a group of users that was representative of typical Twitter users. We identified a sample of 250 control users.

We determined gender for the sample of Twitter users because numerous studies have identified a relationship between gender and suicide risk [36]; as well, mental health symptoms such as depression and anxiety have a known association with gender [37,38]. Additionally, among individuals with schizophrenia, mortality due to suicide is higher in men than in women [2]. We employed a stepwise process for coding each Twitter user’s gender as male, female, or unknown/insufficient data. Two researchers independently used these codes beginning with each Twitter user’s username, followed by profile name, profile description, profile photo, and then tweets. Both researchers then reviewed their final gender codes for each Twitter user to ensure consistency and to resolve any disagreements.

We also extracted several characteristics for the Twitter users included in this study. This involved collecting metadata from the Twitter users’ accounts, including total number of tweets, tweets per day (ie, total tweets divided by days active), tweets in last 200 days, number of friends, number of followers, favorites per day, and number of days the account has been active. We also measured each Twitter user’s impact, which is calculated as a *followers-to-friends* ratio where the user’s number of followers is divided by their number of friends [39]. This serves as a measure of impact and influence on Twitter because a higher ratio means that a user has many people who follow their account, but that they follow few other users’ tweets [39].

In our final sample, included in the analyses reported here, we had a total of 203 Twitter users who self-identified has having schizophrenia and 173 control users. The final number of users changed because some accounts became inaccessible (ie, private, deleted, banned, or deactivated) or were inactive (ie, no posts during the 200-day study period) at the time of data collection.

### Tweets With Suicide Terms

We retrieved all tweets posted during the 200-day period from the Twitter users included in this study. Within this collection of users’ tweets, we identified only tweets that contained the keywords *suicide* or *suicidal*. Prior studies have shown that there are a variety of terms used on social media that may be indicative of suicide risk [8,40]. The term *suicide* is frequently contained in suicide-related conversations [8,41]. Therefore, we intentionally limited our search to these two terms to improve the certainty that the discussion content captured in this study was explicitly referring to suicide. We also considered this important because online discussions about suicide have been correlated with actual suicide risk. For example, a study from Japan showed that statements specifically mentioning the term *suicide* on Twitter were significantly associated with suicidal ideation and behavior [42]. In addition to searching for suicide-related terms, we also selected keywords for other mental health symptoms that are known risk factors for suicide [43]. These include the following terms: *depression*, *depressed*, *anxiety*, and *anxious*.

### Timing of Tweets With Suicide Terms

To examine whether there were potential differences in the timing of tweets containing the terms *suicide* or *suicidal* between groups, we performed an analysis of tweet timing. This involved converting the time-of-day data for tweets containing the terms *suicide* or *suicidal* to the Twitter users’ local time zones, and then classifying these tweets into the following time intervals based on a 24-hour clock: 00:00-05:59, 06:00-11:59, 12:00-17:59, and 18:00-23:59. It was possible to perform this analysis of tweet timing for only the subset of Twitter users’ tweets with available universal time code (UTC) offset data. The UTC offset is only available for a tweet when users choose to include their local time zone in their account settings.

### Statistical Analyses

We used *t* tests to compare continuous variables and chi-square tests to compare categorical variables between the group of Twitter users with schizophrenia and the control users. We used logistic regression models controlling for gender to compare both groups on the binary outcomes of whether or not users posted a tweet containing suicide terms (ie, *suicide* or *suicidal*). We then used Pearson correlations to assess whether tweeting about other mental health terms (eg, depression or anxiety) would be associated with posting a tweet with a suicide term. All analyses were performed with the Python programming language and Stata version 14.0 (StataCorp LLC).

## Results

### Sample Characteristics

During the 200-day study period from January 2016 to July 2016, we collected a total of 1,544,122 tweets, with 819,491 tweets (53.07%) posted by the Twitter users with schizophrenia (N=203) and 724,631 tweets (46.93%) posted by the control users (N=173). Characteristics between the two groups were generally similar. Twitter users with schizophrenia posted a comparable number of tweets per day (mean 21.10, SD 58.50) as the control users (mean 20.80, SD 34.30). The *followers-to-friends* ratio among Twitter users with schizophrenia (mean 7.17, SD 52.40) was also similar to that of control users (mean 2.56, SD 6.33). Only gender differed significantly between groups, where a larger proportion of Twitter users with schizophrenia (93/203, 45.8%) were identified as male compared to control users (57/173, 32.9%; χ^2^_2_=8.1, *P*=.02).

### Tweets About Suicide

Differences in tweets about suicide between groups are listed in Table 1. Twitter users with schizophrenia tweeted significantly more about suicide (mean 7.10, SD 15.98) compared with control users (mean 1.89, SD 4.79; *t*_374_=-4.13, *P*<.001). Among the 203 Twitter users with schizophrenia, 113 (55.7%) posted a total of 1441 tweets about suicide (mean 12.75, SD 19.69) compared to 65 out of 173 (37.6%) users in the control group who tweeted about suicide 327 times (mean 5.03, SD 6.75). In a logistic regression model adjusting for gender, Twitter users with schizophrenia showed significantly greater odds of tweeting about suicide compared with control users (odds ratio 2.15, 95% CI 1.42-3.28).

### Timing of Tweets About Suicide

In our total sample of Twitter users, 71.0% (267/376) had available time zone data. There was no significant difference in availability of time zone information between Twitter users with schizophrenia (137/203, 67.5%) and control users (130/173, 75.1%). There were no differences in the proportions of tweets about suicide during each time interval between the Twitter users with schizophrenia and control users. In general, and as presented in Table 2, both groups appeared to post a comparable proportion of their tweets about suicide at each time point.

**Table 1:** Tweets containing terms about suicide among Twitter users with schizophrenia and control users

Suicide terms	Control Twitter users (N=173)			Twitter users with schizophrenia (N=203)			*t*_374_	*P* value^a^
	Tweets, n	Tweets per user, mean (SD)	Users who tweeted, n (%)	Tweets, n	Tweets per user, mean (SD)	Users who tweeted, n (%)		
Suicide	266	1.54 (3.81)	60 (34.7)	1095	5.39 (12.88)	106 (52.2)	-4.09	<.001
Suicidal	62	0.36 (1.93)	23 (13.3)	367	1.81 (4.60)	66 (32.5)	-3.82	.006
Tweets with any suicide terms^b^	327	1.89 (4.77)	65 (37.6)	1441	7.10 (15.94)	113 (55.7)	-4.60	<.001

^a^*P* values calculated using *t* tests for the difference in mean (SD) tweets containing suicide or suicidal terms between Twitter users with schizophrenia and control users.

^b^This category also includes tweets that contain both the terms *suicide* and *suicidal*.

**Table 2:** Timing of tweets containing the terms *suicide* and *suicidal* among Twitter users who self-identify as having schizophrenia compared to control Twitter users

Time interval	Proportion of tweets among control Twitter users containing the terms *suicide* and *suicidal* (N=286), n (%)	Proportion of tweets among Twitter users with schizophrenia containing the terms *suicide* and *suicidal* (N=1101), n (%)	χ^2^_1_	*P* value^a^
00:00-05:59	28 (9.8)	145 (13.17)	2.4	.12
06:00-11:59	71 (24.8)	260 (23.61)	0.2	.67
12:00-17:59	94 (32.9)	376 (34.15)	0.2	.68
18:00-23:59	93 (32.5)	320 (29.06)	1.3	.26

^a^*P* values calculated using chi-square tests.

### Predictors of Tweets About Suicide

**Figure 1:**
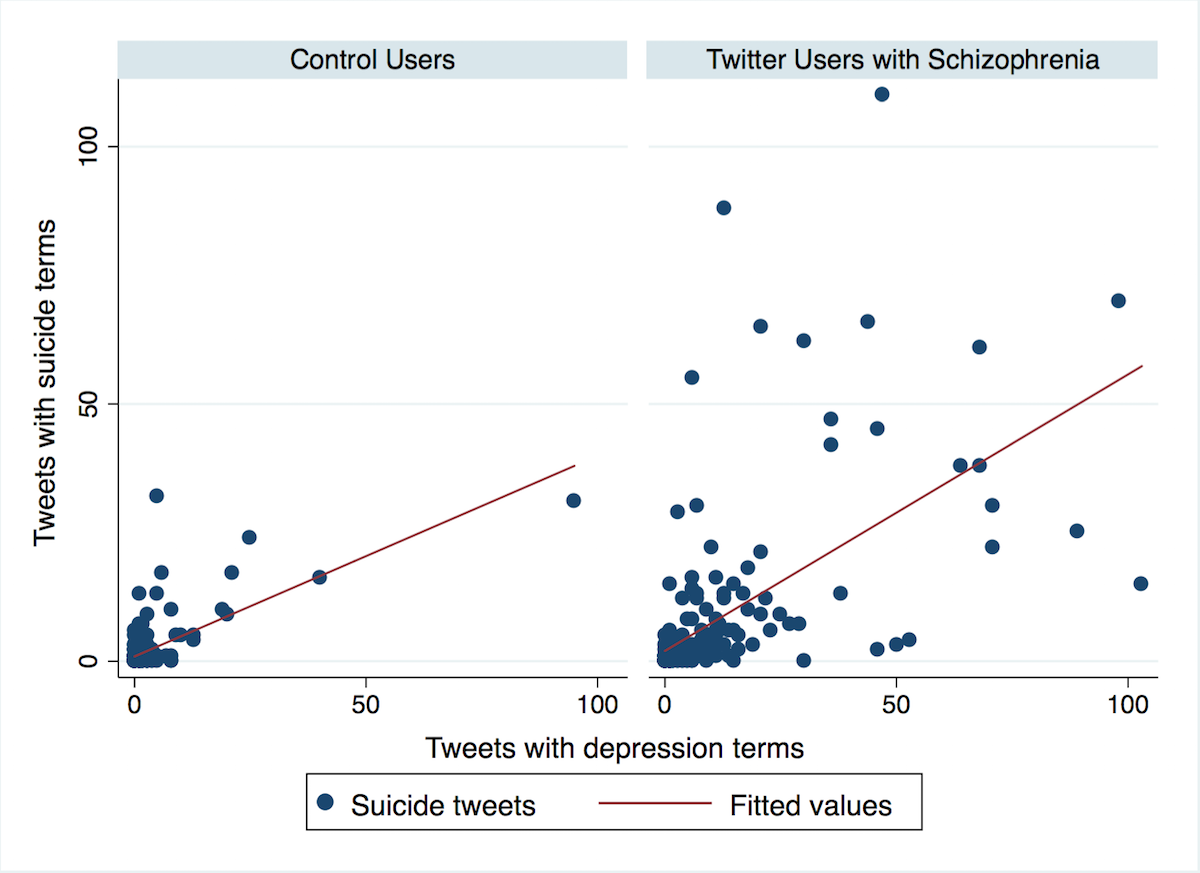
Association between tweets containing terms about suicide and tweets containing terms about depression among Twitter users with schizophrenia and control users.

**Figure 2:**
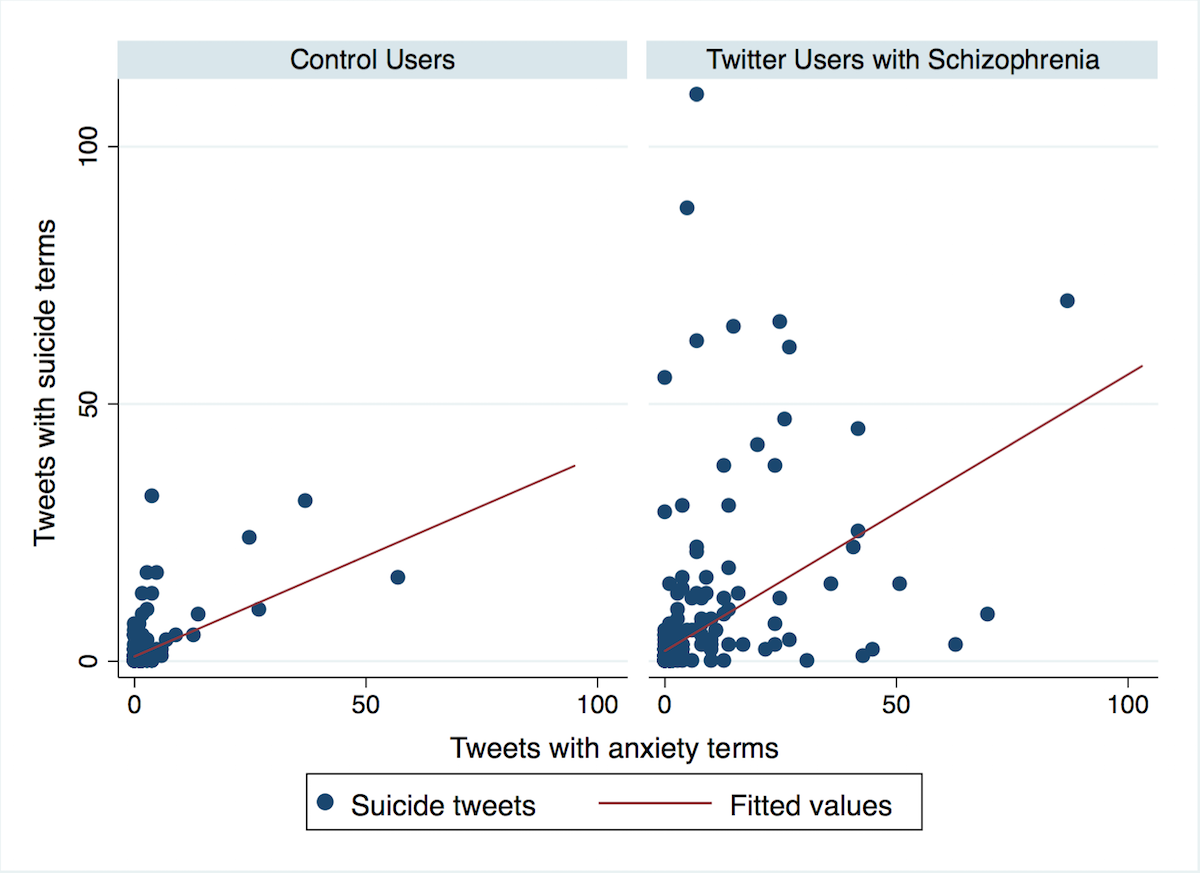
Association between tweets containing terms about suicide and tweets containing terms about anxiety among Twitter users with schizophrenia and control users.

Across both groups, frequency of tweets containing suicide terms was significantly associated with tweets about depression (*r*=0.62, *P*<.001) and with tweets about anxiety (*r*=0.45, *P*<.001). Correlations between suicide tweets and depression tweets, and between suicide tweets and anxiety tweets, are illustrated for each group in Figure 1 and Figure 2, respectively. Regarding Figure 1, Pearson correlations were calculated for the association between tweets containing suicide terms and tweets containing depression terms for the Twitter users with schizophrenia group (*r*=0.60, *P*<.001) and the control users group (*r*=0.70, *P*<.001). Regarding Figure 2, Pearson correlations were calculated for the association between tweets containing suicide terms and tweets containing anxiety terms for the Twitter users with schizophrenia group (*r*=0.40, *P*<.001) and the control users group (*r*=0.62, *P*<.001).

## Discussion

### Principal Findings

The impact of suicide among individuals with schizophrenia is devastating. There is a well-established association between diagnosis of schizophrenia and increased risk of death from suicide, suicide attempts, and suicidal ideation [22]. Among all causes of death observed in persons with schizophrenia, suicide accounts for the highest mean years of potential life lost per death [2] and represents a major contributor to the dramatically shortened life expectancy observed in this patient group [44]. With increasing emphasis on the potential use of social media for suicide prevention [5], our findings offer an important contribution to the literature by (1) demonstrating the feasibility of identifying conversations about suicide among Twitter users who self-identify as having schizophrenia; (2) highlighting that Twitter users who self-identify as having schizophrenia are significantly more likely to talk about suicide compared to a control sample of Twitter users, which parallels trends observed in offline settings; and (3) demonstrating that the frequency of conversations about suicide on Twitter correlated significantly with discussions about depression and anxiety, another trend that is consistent with established data. Therefore, our findings represent an early indication that popular social media may be a valuable platform for monitoring discussion about suicide among persons who self-identify as having schizophrenia. Furthermore, our study offers a unique contribution to an emerging area of research aimed at using social media to support the detection of schizophrenia and for identifying individuals at risk of psychosis [17-20]. As this research using social media continues to evolve, there may be opportunities to explore online conversations on social media for simultaneously monitoring risk of psychosis and risk of suicide.

It is important to draw the connection between our findings reported here and suicide trends observed among persons with schizophrenia in the “offline” world. While it was not possible in this study to determine whether conversations about suicide among Twitter users who self-identify as having schizophrenia correlate with actual suicidal ideation or intent, our findings add to an increasing number of studies demonstrating that data captured from popular social media correlates strongly with data collected offline. For example, a recent study from Japan found that increases in conversations about suicide on Twitter were associated with increases in the number of actual suicide deaths over time [7]. Similarly, another study found that variation in Twitter conversations about suicide paralleled geographic distribution of real-world suicide rates in the United States [8]. Together, these studies suggest that discussion about suicide on Twitter may be an indicator of true suicide risk.

Our study offers an additional important contribution by demonstrating strong correlations between conversations about suicide and conversations about depression or anxiety in both Twitter users who self-identify as having schizophrenia and control users. This is consistent with prior studies that have demonstrated that depression is a significant risk factor for suicide [45], as well as research showing that symptoms of anxiety also correlate with suicide risk [46,47]. For instance, a recent study following Reddit users over time found that those who proceed to discuss suicidal ideation were more likely to have previously posted content online reflecting various psychological and behavioral states, including increased anxiety, when compared to users who do not proceed to discuss suicidal ideation [48]. Therefore, our findings add to this recent work by further emphasizing the association between online discussion about mental health and suicidal ideation among social media users [48].

### Ethical Considerations

Key considerations with using social media as a tool for monitoring conversations about suicide pertain to broader ethical challenges with using publicly available online data, as well as the need to carefully weigh threats to safety and privacy against benefits gained by using novel approaches to study suicide in this way. For example, we only examined publicly available data in this study, yet many individuals who openly share sensitive personal information on social media do so without fully realizing how this information will be used, who will use it, and in what ways it could potentially result in harm [33]. While the Twitter users whose data we examined in this study self-identified as having schizophrenia, it is important to recognize these individuals as belonging to a vulnerable group because disclosure of mental illnesses like schizophrenia is associated with societal stigma and risk of discrimination. Therefore, we removed usernames and all identifying information from users’ content analyzed in this study; in addition, we did not report any quoted text, as this is a recommended approach for balancing the privacy of Twitter users with the aims of research [33]. Interestingly, monitoring conversations about suicide on Twitter may uncover unanticipated crises or urgent health risks, highlighting the need to consider how best to respond to individuals potentially at risk of suicide and ensuring that these individuals receive access to necessary professional support [5]. Importantly, researchers have emphasized that the public nature of social media platforms like Twitter may yield valuable opportunities for intervention and supporting suicide prevention [5]. Future research is needed to expand on our current exploratory work for considering how social media platforms could be leveraged to support suicide prevention and early intervention among individuals with schizophrenia.

Connecting and interacting with others is a key attribute of social media platforms, which may expose individuals to unforeseen influences from others and possible risks [5]. Alternatively, social media platforms may allow individuals to seek peer-to-peer support, as has been previously observed among individuals with mental illness [15,49]; these platforms may also enable access to a valuable social support network, which is known to be associated with reduced suicide risk among persons with schizophrenia [50]. It is also necessary to carefully develop procedures and protocols that account for these ethical challenges and include strategies for risk management, as well as comprehensive approaches for protecting social media users’ safety while balancing the potential risk of suicide [51]. Ongoing efforts are needed to determine how to be mindful of potential ethical concerns while identifying novel approaches for supporting individuals with schizophrenia who may be at risk of suicide. This is especially important because there remains much uncertainty regarding ideal strategies for preventing suicide among vulnerable patient groups, such as individuals with schizophrenia [50].

### Limitations

This was an exploratory study; therefore, caution is warranted when interpreting these findings. Several limitations should be considered. First, without access to psychiatric histories, it was not possible to confirm clinical diagnoses for the Twitter users who self-identified as having schizophrenia in this study. Additionally, these Twitter users likely differ from individuals with schizophrenia who do not disclose their illness online or who do not use social media. It is critical for future research to link content of discussions captured from social media with established clinical criteria to further support the generalizability of digital mental health detection methods [32]. Second, we were not able to collect demographic data from the Twitter users included in this analysis. National surveys indicate that Twitter users are typically younger than the general population [21], which is important when considering the implications of using social media for monitoring suicide risk in young persons; however, we were unable to determine the age of the users included in this study. This is a common challenge in public health research using social media [52]. In general, Twitter users tend to be young, have college degrees, and come from higher socioeconomic status groups [21]. A recent study showed that Twitter users with schizophrenia appeared to have high levels of education and, thus, may have fewer cognitive or functional limitations when compared to individuals with schizophrenia who do not use social media [16]. As a result, our findings likely do not generalize to individuals who do not use social media.

Third, we employed a convenience sampling approach to generate the group of Twitter users who self-identify as having schizophrenia as well as the control group. This sampling method further limits generalizability of these findings. Additionally, because the control group consisted of a randomly generated sample of Twitter users, we cannot rule out the possibility that individuals in this group could also have had a schizophrenia spectrum disorder, though the chance of this is low as schizophrenia prevalence is roughly 1% [53]. However, Twitter users in the control group could have had other types of mental illness associated with increased risk of suicide. Fourth, only a limited number of search terms for suicide were used in this study and it was not possible to confirm whether use of these terms referred to actual suicide risk or behaviors. Our selection of the terms *suicide* and *suicidal* was aimed at improving certainty that the online conversations captured in this study were in fact related to suicide; however, it is possible that use of these may have been in the context of suicide prevention or other unrelated topics. Future research will need to explore the context of social media conversations about suicide to determine whether it relates to help-seeking, sharing experiences, or offering support, and how this contributes to reduced or heightened suicide risk. Additionally, there are several other terms that appear to reflect suicide risk on social media [8], suggesting that the current analysis may underestimate the frequency of online discussion related to suicide among this sample of Twitter users.

### Conclusions

Our study takes preliminary steps toward demonstrating that Twitter users with schizophrenia appear to openly discuss suicide-related topics on Twitter and that these discussions are strongly correlated with conversations about common mental health symptoms known to be associated with actual suicide risk. This is an initial step toward informing the use of social media for monitoring suicide risk among people with serious mental illnesses such as schizophrenia. The need for effective approaches for detecting suicide risk remains a significant public health challenge [54]. The important opportunities to use social media for detecting and responding to suicide risk should not be missed. Going forward, it will be essential to weigh these benefits with potential ethical considerations related to individual privacy and ensuring adequate and timely responses to distressing content posted online. Therefore, future efforts are necessary to expand on our work presented here to develop and evaluate the use of social media for detecting suicide risk among individuals with schizophrenia, while seeking to leverage these popular online platforms for supporting suicide prevention efforts.

## Abbreviations

UTC: universal time code
